# Effect of Heat-Moisture and Annealing Treatments on the Physicochemical Properties and Enzymatic Hydrolysis of Quinoa Starch

**DOI:** 10.3390/foods15193567

**Published:** 2026-10-08

**Authors:** Yuxue Zheng, Xiaoning Yu, Shaoyun Wang

**Affiliations:** School of Biological Science and Engineering, Institute of Food and Marine Bio-Resources, Fuzhou University, Fuzhou 350108, China

**Keywords:** quinoa starch, heat-moisture treatment, annealing treatment, physical properties, digestion

## Abstract

Quinoa starch shows promise for low-glycemic foods, but its rapid digestibility and limited processability constrain its application. While heat-moisture (HMT) and annealing (ANN) treatments are widely used for starch modification, their systematic comparison on quinoa starch across moisture and temperature remains unreported. This study therefore investigated the effects of HMT and ANN on the physicochemical properties and digestibility of quinoa starch. Both treatments changed the starch surface structure, weakened the long-range and short-range ordered structures and impaired the freeze–thaw stability of quinoa starch. HMT reduced starch peak viscosity as moisture content increased, while ANN showed a temperature-dependent response: low-temperature ANN (30–40 °C) increased peak viscosity, whereas high-temperature ANN (50–60 °C) decreased it. In vitro digestion results showed that 15% moisture HMT and 50 °C ANN reduced rapidly digestible starch (RDS) to 40.77 ± 0.96% and 48.57 ± 0.90%, respectively. The cumulative intestinal digestion at 120 min decreased from 69.33% (native starch) to 57.06–63.97% for HMT-treated samples and to 58.20–67.29% for ANN-treated samples. These findings provide a practical basis for designing quinoa starch-based foods with tailored digestibility.

## 1. Introduction

Quinoa is a dicotyledonous pseudocereal that belongs to the *Chenopodiaceae* family and is rich in amino acids, high-quality protein and many other bioactive substances such as polyphenols and insoluble dietary fiber [1,2]. In addition, starch is also one of the most important components in quinoa, with a content of 50–60% [3]. The amylopectin content of quinoa starch is relatively low, approximately 2.7–16.9%, and the particle size is about 10–30 μm, making it a typical small-grain starch [4]. The crystal form of quinoa starch is type A, with a relative crystallinity of approximately 25%, and the proportion of short B chains in the amylopectin is higher, resulting in limited flexibility of the external chain, which is not conducive to the orderly arrangement of the double helix in the crystalline region [5].

Hydrothermal treatment (HMT) and annealing treatment (ANN) are two widely used hydrothermal treatment methods for cereal starch [6]. HMT treatment is a starch modification method conducted at high temperatures (90–120 °C) with a controlled system moisture content of 10–30% (*w*/*w*) for 15 min to 16 h [7,8]. Typically, moisture is adjusted by adding distilled water to native starch, which is then equilibrated at room temperature for 24 h, and the treatment is performed in sealed, pressure-resistant containers. In contrast, ANN treatment is performed under high-moisture and low-temperature conditions. Specifically, starch granules are incubated in moderate (40–55%, *w*/*w*) or excessive (>60% *w*/*w*) moisture environments at temperatures between the glass transition temperature and gelatinization temperature (40–60 °C) for periods ranging from several minutes to several days [7,9]. It should be noted that HMT and ANN represent fundamentally different modification regimes: HMT operates under limited moisture and high temperature, whereas ANN operates under excess moisture and sub-gelatinization temperature. These differences in moisture availability and thermal energy input inherently lead to distinct structural reorganization pathways, and their effects should be compared with this distinction in mind.

Previous studies have proven that HMT and ANN can effectively regulate the physical characteristics of starch, including its surface structure, crystal type, molecular weight, thermal properties, and digestibility [8,10]. These property changes are generally attributed to the combined effects of thermal energy and moisture, which promote molecular chain mobility within the starch granules, leading to partial disruption of hydrogen bonds and double helices, and subsequent reorganization of the multi-scale structure. Such structural reorganization can alter the accessibility of starch to digestive enzymes and thereby modulate its digestion characteristics. The specific structural changes, however, depend on the starch source and treatment conditions [11,12].

At present, HMT and ANN have been widely studied and applied to regulate starch digestibility. Xie et al. found that rice starch treated with HMT (30% moisture content, heated at 110 °C for 4 h) had a higher resistant starch (RS) content [13]. The in vitro digestibility test results showed that the HMT (120 °C/24 h, 30% moisture) and HMT-ANN (HMT, 120 °C/24 h, 30% moisture; ANN, 50 °C/24 h, 70% moisture) treatments increased the RS and slowly digestible starch (SDS) content of bitter vetch (*Vicia ervilia*) starch by approximately 1.5 times and 4 times, respectively [14]. Furthermore, in another study, Zheng et al. subjected potato, corn, and pea starch to ANN treatments at 50 °C for 24, 48, and 72 h respectively. The results showed that as the annealing time extended to 72 h, the SDS and RS contents in potato starch increased; in contrast, the RS contents of corn starch and pea starch did not change after the 72 h annealing treatment. This might be due to the differences in their internal structures (such as crystal types) [15]. These results indicated that starch varieties and treatment conditions were important factors that affected starch digestibility under HMT and ANN treatments [16].

As summarized above, previous studies have shown that HMT and ANN treatments could change the structure of starch, thus affecting the digestion rate of starchy foods [11]. However, these studies have largely examined HMT and ANN separately, and the treatment conditions covered a limited range. A systematic comparison of HMT and ANN treatments on the same quinoa starch across a gradient of moisture levels and temperatures has not been reported, and the quantitative relationship between treatment conditions and digestibility outcomes remains unclear. Therefore, the present study explored the effects of HMT and ANN treatments on the gelatinization properties, microscopic morphology, semi-crystalline crystal form, thermal stability, in vitro digestive properties and other physical properties of quinoa starch and systematically revealed the mechanism of the regulation of the digestibility of multigrain starch by heat-moisture and annealing treatment. These findings provide a practical basis for selecting HMT moisture levels and ANN temperatures to tailor the digestibility of quinoa starch, supporting its application in low-glycemic foods.

## 2. Materials and Methods

### 2.1. Materials

Quinoa flour was purchased from Gansu Qilian Farm Organic Agriculture Development Co., Ltd. (Jinchang, China). Porcine pancreatic alpha-amylase (from porcine pancreas, CAS: 9000-90-2, ≥5 U/mg solid), alpha-amyloglucosidase (from *Aspergillus niger*, CAS: 9032-08-0, 100000 U/mL), and invertase (from bread yeast, CAS: 9001-57-4, ≥300 U/mg solid) was purchased from Sigma-Aldrich Company (St. Louis, MO, USA). The glucose assay kit was obtained from Megazyme International (Bray, Co. Wicklow, Ireland), and other analytical-grade reagents were bought from Aladdin Co., Ltd. (Shanghai, China).

### 2.2. Preparation of Quinoa Starch

Quinoa flour was soaked in an NaOH solution (0.2%, *w*/*w*) for 5 h with a ratio of 1:5 (g/mL). And then the mixed liquid was centrifuged at 4000 rpm for 10 min, the supernatant was removed, the sediment was repeated washing and centrifuged with the same condition for 3 times, and dried in a 40 °C oven, after that the samples were sieved through a 100-mesh sieve and stored at room temperature in a desiccator for future use. The starch extraction was performed in three independent batches, and the final values are reported as the mean of these batches. The starch yield was 32.3%, the final starch content was 88.13%, moisture content was 8.5%, residual protein was 0.42%, lipid content was 0.35%, and ash content was 0.18%. These values are consistent with those reported for quinoa starch extracted by alkaline methods [17].

### 2.3. HMT Treatment of Quinoa Starch

The method of HMT treatment of quinoa starch followed the method of Wu et al. [18]. The moisture levels of 15%, 20%, 25%, and 30% were selected because they cover the conventional HMT moisture range (10–30%) and allow evaluation of the moisture-dependent threshold effect on quinoa starch. Distilled water was added to adjust the starch samples to the target moisture content on a wet basis, calculated as:

water added (g) = starch weight (g) × (target moisture − initial moisture)/(100 − target moisture).

After sealing, allowed them to equilibrate at room temperature for 24 h to reach moisture equilibrium. The samples were then heated at 100 °C for 2 h under limited moisture conditions (15–30% wet basis), representing a high-temperature, low-moisture modification regime. All starch samples were prepared in three independent samples. Each sample was subjected to the same treatment conditions but prepared separately from the same starting quinoa starch. The HMT-treated quinoa starch samples were labeled as HMT-15, HMT-20, HMT-25, and HMT-30.

### 2.4. ANN Treatment of Quinoa Starch

The ANN treatment of quinoa starch was conducted according to the method of Zheng et al. [15] with modifications. The temperature range of 30 °C–60 °C was selected because it spans from well below to near the gelatinization onset temperature of quinoa starch (61.4 °C), allowing evaluation of the temperature-dependent transition from annealing to partial gelatinization. Briefly, 12 g of quinoa starch was accurately weighed and mixed thoroughly using a magnetic stirrer with 60 mL of distilled water in a glass beaker, corresponding to a moisture content of approximately 83% (wet basis). This high water-to-starch ratio was chosen to ensure excess moisture conditions, which are required for conventional ANN treatment. The beaker was then subjected to a water bath at different temperatures (30 °C, 40 °C, 50 °C, 60 °C) for 24 h. It should be noted that ANN-60 was performed at a temperature close to the gelatinization onset temperature of native quinoa starch (61.4 °C), and this treatment may represent a near-gelatinization condition rather than conventional annealing. Subsequently, the sample was dried in a drying oven at 40 °C for 12 h, followed by grinding and further stored in sealed bags for subsequent analysis. All starch samples were prepared in three independent samples. Each sample was subjected to the same treatment conditions but prepared separately from the same starting quinoa starch. The samples were labeled as ANN-30, ANN-40, ANN-50, and ANN-60.

### 2.5. Characterization of Starch Surface Microstructure

The surface structure of the starch samples was observed with a Gemini 300 Scanning Electron Microscope (Carl Zeiss GmbH, Oberkochen, Germany). A circular tray was affixed with conductive glue, and the starch samples were evenly spread on the conductive glue. After ion sputtering and gold spraying, the samples were placed in a scanning electron microscope, and the starch morphologies were observed at 20,000 magnifications.

### 2.6. Freeze-Thaw Stability Determination

The freeze–thaw stability of samples was determined according to the method of Zheng et al. with some modifications [19]. The details were as follows, 1 g of starch sample and 16 mL of distilled water were placed in a 100 °C water bath for 30 min; the mixture was then placed in a pre-weighed 10 mL centrifuge tube, stored at −20 °C for 24 h, and then thawed at room temperature for 4 h. During each freeze–thaw cycle, the tube was centrifuged at 1500 rpm for 15 min and the remaining water was drained. The freeze–thaw cycles were repeated three times. The water evolution rate was calculated as follows:

Water evolution rate (%) = (W/M) × 100

where W was the mass of precipitated water, g; M was the total sample mass, g. A higher water evolution rate indicated greater syneresis and poorer freeze–thaw stability, whereas a lower water evolution rate indicated better freeze–thaw stability.

### 2.7. Thermal Properties Determination

A differential scanning calorimeter (DSC) was applied to determine the thermal properties of starch before and after HMT and ANN according to the method of Zhang et al. [20]. The thermal properties were determined using a Mettler-Toledo differential scanning calorimetry (Schwerzenbach, Switzerland). During the heating procedure, a blank aluminum crucible was used as a control and the sample was heated at a rate of 10 °C/min, from 25 °C to 110 °C, then cooled to 30 °C. In this process, the initial temperature (T_o_), peak temperature (T_p_), end temperature (T_c_) and enthalpy value (∆H) of starch sample gelatinization were recorded.

### 2.8. Gelatinization Properties Determination

An RVA 4500 Viscosity Measuring Instrument (PerkinElmer Pty Ltd., Melbourne, Australia) was used to measure the gelatinization curves of quinoa starch according to the method of Zheng et al. [15]. The starch sample (2.58 g, dry basis) was accurately weighed and mixed with distilled water to a total weight of 28.00 g. The temperature program was set as follows: the slurry was held at 50 °C for 1 min, heated to 95 °C at a rate of 12 °C/min, held at 95 °C for 5 min, cooled to 50 °C at a rate of 12 °C/min, and held at 50 °C for 1 min. Pasting parameters were recorded from the pasting profile, including peak viscosity (PV), trough viscosity (TV), breakdown (BD), final viscosity (FV), setback (SB), and pasting temperature (PT).

### 2.9. Determination of Long-Range Ordered Structure of Quinoa Starch

The crystallinity and relative crystallinity of starch were determined by an X-ray diffractometer according to the method of Zheng et al. [21] with Cu Ka target radiation (λ = 0.15406 nm). The instrument was operated at 40 kV and 40 mA. Freeze-dried starch samples were scanned at 2θ = 4–40° with a step size of 0.04° and a scanning speed of 0.05°/s. The relative crystallinity (RC) was calculated as the ratio of the area of crystalline peaks to the total area of the diffraction pattern using MDI Jade 6.5 software (Materials Data Inc., Livermore, CA, USA).

### 2.10. Determination of Short-Range Ordered Structure of Quinoa Starch

Fourier transform infrared spectroscopy (FT-IR, Nicolet 5700, Thermo Nicolet Co., Waltham, MA, USA) was applied to measure samples at room temperature. The samples were mixed with KBr at a ratio of 1:100 (*w*/*w*) ground, and pressed into sheets. The samples were analyzed using a Fourier Transform spectrometer for determination. The scanning range was 4000–400 cm^−1^ and the resolution was 4 cm^−1^. Using spectral grade KBr as blank reference, the infrared spectrum was obtained by averaging 32 scanns. Spectral processing was performed using OMNIC software (Thermo Nicolet). The R1047/1022 ratio was calculated as the ratio of absorbance at 1047 cm^−1^ to that at 1022 cm^−1^ using the baseline-corrected and normalized spectra. The reported R1047/1022 values are the mean ± standard deviation of the three independently prepared samples.

### 2.11. Digestive Properties

#### 2.11.1. Enzymatic Analysis of Quinoa Starch

Enzymatic hydrolysis studies were performed to analyze the resistance of quinoa starch to hydrolysis according to the method of Chai et al. [22] with some modifications. Briefly, starch sample (0.25 g, dry basis) was mixed with 12.5 mL of acetate buffer (pH 5.2, 0.1 mol/L) and gelatinized at 90 °C for 20 min with stirring. After cooling to 37 °C, 0.75 mL of an enzyme solution containing α-amylase (120 U/mL) and α-amyloglucosidase (240 U/mL) was added. The mixture was incubated at 37 °C for 2 h with magnetic stirring. Aliquots (0.5 mL) were taken at 0, 20, and 120 min and immediately mixed with 2.5 mL of ethanol to inactivate the enzymes. After the mixed solution was centrifuged at 4000 r/min for 10 min, the glucose content in the supernatant was determined using a GOPOD glucose assay kit (Megazyme, Bray, Ireland) with a glucose standard curve (0–1.0 mg/mL). Absorbance was measured at 510 nm, and glucose concentration was calculated from the standard curve. The contents of RDS, SDS and RS were calculated according to Formulas (1)–(3):

RDS = 0.9 × (G20 − G0)(1)

SDS = 0.9 × (G120 − G20)(2)

RS = TS − RDS − SDS(3)

where G0, G20 and G120 represent the glucose content produced by hydrolysis at 0, 20 and 120 min, TS represents the total starch content, and 0.9 is the conversion coefficient.

#### 2.11.2. In Vitro Gastrointestinal Simulation Digestion

The gastrointestinal digestion of starch samples was performed based on the method of Liu et al. [23] with some modifications. Simulated gastric fluid (SGF) and simulated intestinal fluid (SIF) were prepared according to the same reference. Starch samples (500 mg, dry basis) were mixed briefly with 5 mL of deionized water and gelatinized at 95 °C for 30 min, then cooled to 37 °C. For the gastric phase, 4 mL of SGF (at pH 3.0, containing pepsin at a final activity of 2000 U/mL) was added, and the mixture was incubated at 37 °C while shaking at 150 rpm for 120 min. For the intestinal phase, the pH was adjusted to 6.8 with 1 M NaOH, and 5 mL of SIF (containing pancreatin at 200 U/mL and bile salts at 20 mM) was added. The mixture was further incubated at 37 °C while shaking at 150 rpm for 180 min. Aliquots (200 μL) were collected at 0, 20, and 120 min during the intestinal phase and immediately mixed with 1800 μL of 80% (*v*/*v*) ethanol to inactivate the enzymes. After centrifugation at 10,000 rpm for 10 min, the supernatant was diluted 1:3 with deionized water, and the glucose content was determined using a glucose kit.

### 2.12. Statistical Analysis

Within each independently prepared sample, measurements were performed in triplicate (three technical replicates), and the mean of the three technical replicates was used as one data point. The results were reported as the mean ± standard deviation of the three independently prepared samples. One-way analysis of variance (ANOVA) was performed across all groups using GraphPad Prism version 8.0 (GraphPad Software, Boston, MA, USA), followed by Tukey’s post hoc test for pairwise comparisons. Statistical significance was set at *p* < 0.05.

## 3. Results and Discussion

### 3.1. Surface Microstructure Characterization

**Figure 1:**
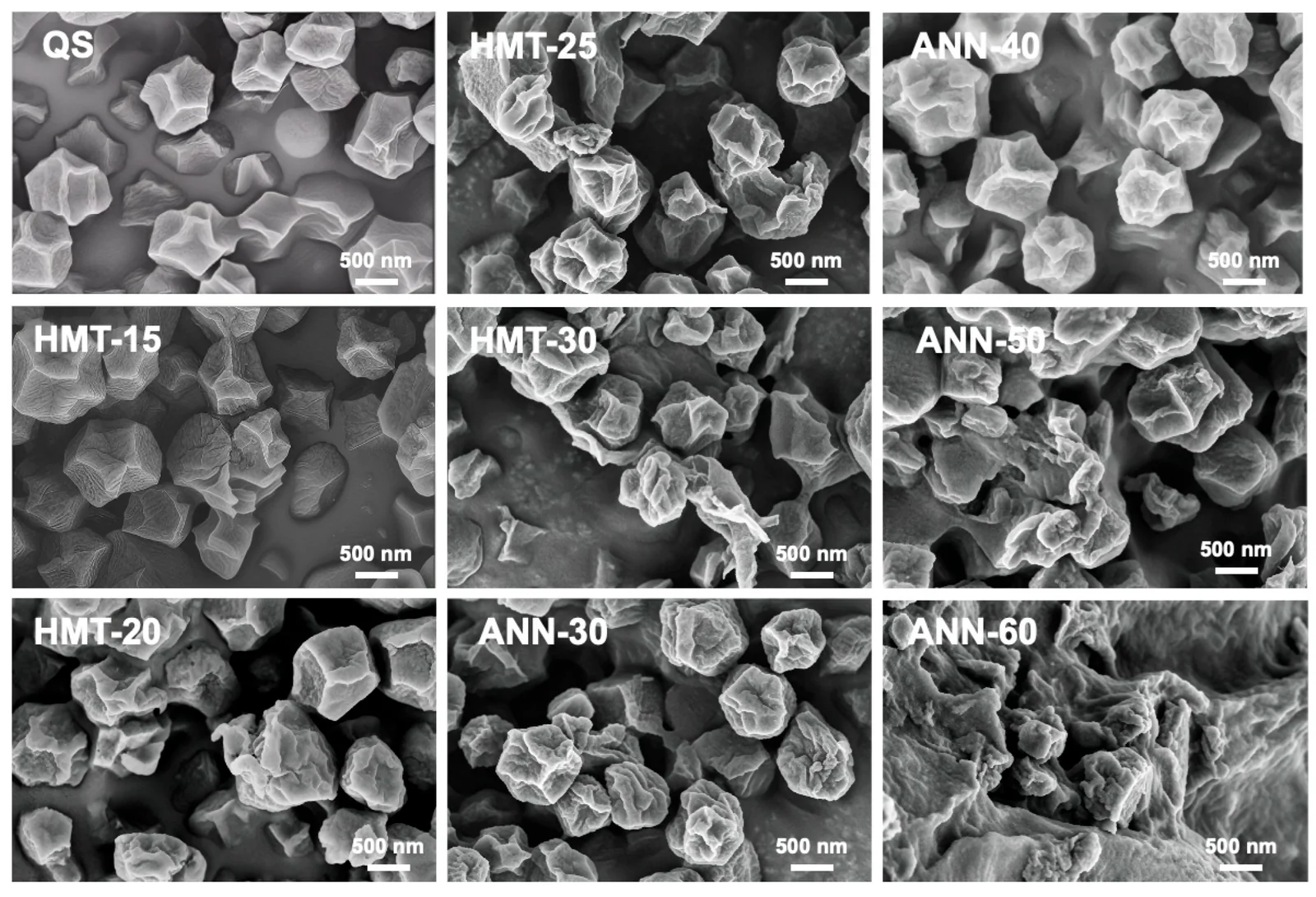
Surface structure of quinoa starch treated with HMT and ANN, scale bar = 500 nm.

Figure 1 shows the morphologies of native quinoa starch and quinoa starch modified by HMT (HMT-15, 20, 25, 30) and ANN (ANN-30, 40, 50, 60) treatments. Native quinoa starch exhibited irregular polygons with some voids or cracks on the surface. HMT modification did not change granule morphology at 15% and 20% moisture, while increased cracking was observed in HMT-25 and HMT-30. The increase in cracks observed on HMT-25 and HMT-30 was consistent with the results of Dubey et al. [24]. They reported that heating quinoa starch at a moisture content of 22% and a temperature of 110 °C for 4 h could cause significant surface damage, including cracks, holes, and irregular particle shapes, which was attributed to partial gelatinization under limited moisture conditions and the generation of internal stress [25].

For ANN, ANN-30 remained unchanged, ANN-40 showed some cracks, and ANN-50 exhibited breakage and clustering. Notably, ANN-60 resulted in extensive apparent granule disruption and aggregation, with the original polygonal structure largely lost. This was consistent with ANN-60 being performed at 60 °C, close to the gelatinization onset temperature of native quinoa starch (61.4 °C), under excess moisture for 24 h, which likely induced partial gelatinization. The cracks in ANN-40 and ANN-50 may be attributed to amylose recombination [26], while granule aggregation in ANN-50 and ANN-60 resulted from heating under sufficient moisture, forming more compact amorphous regions [27].

These morphological differences reflected the distinct processing environments of HMT and ANN. HMT operated under limited moisture (15–30%) and high temperature (100 °C) for 2 h, primarily affecting the granule surface and amorphous regions. ANN operated under excess moisture (almost 83%) and sub-gelatinization temperature (30–60 °C) for 24 h, allowing prolonged hydration and molecular reorganization throughout the granule. Therefore, the observed structural differences between HMT- and ANN-treated samples arise from differences in moisture availability, treatment temperature, and duration.

### 3.2. The Freeze–Thaw Stability of Quinoa Starch

As shown in Table 1, the water evolution rates of quinoa starch differed among treatments. A higher water evolution rate indicated greater syneresis and poorer freeze–thaw stability, whereas a lower water evolution rate indicated better freeze–thaw stability. Native quinoa starch had the lowest water evolution rate (30.19%), demonstrating the best freeze–thaw stability. After HMT and ANN treatments, the water evolution rate increased, indicating decreased freeze–thaw stability. In the HMT group, the water evolution rate increased from 36.40% (HMT-15) to 42.22% (HMT-30) as moisture content increased [28]. In the ANN group, the water evolution rate increased from 31.60% (ANN-30) to 42.92% (ANN-60) as annealing temperature increased. ANN-30 showed no significant difference from native starch (*p* > 0.05), whereas ANN-50 and ANN-60 showed the highest water evolution rates, indicating the poorest freeze–thaw stability. At higher temperatures (50–60 °C), ANN-treated samples showed higher water evolution rates than HMT-treated samples, indicating that high-temperature ANN caused a greater decrease in freeze–thaw stability than HMT [29]. Among all samples, ANN-60 showed the highest water evolution rate (42.92%), indicating the worst freeze–thaw stability. In another study, researchers also reported that ANN treatment reduced the water absorption capacity of foxtail millet starch and attributed those results to the structural disintegration of the starch granules during ANN treatment [30].

### 3.3. Thermal Characteristic Analysis

Table 2 shows the thermal parameters of different quinoa starches, including T_o_, T_p_, T_c_, and ΔH. HMT and ANN treatments substantially influenced these parameters. In the HMT group, ΔH decreased from 3.73 ± 0.46 J/g (QS) to 2.42 ± 0.17 J/g (HMT-25) as moisture content increased, possibly because the original structure of the starch was disrupted during the treatment process. Similar results were reported by Rozendo et al. [31], who found that HMT (25% moisture, 110 °C, 1 h) reduced the ΔH of native quinoa starch, which was attributed to the partial disruption of crystalline regions and the weakening of double-helical order caused by the combined effects of moisture and heat.

In the ANN group, the ΔH increased first and then decreased, with ANN-60 showing the smallest value, indicating that the low temperature of annealing treatment was conducive to strengthening the structure of the double helix or crystallization region, resulting in a more complete and regular crystal structure [32]. The T_o_ value of quinoa starch after treatment increased to different degrees (except ANN-30 and ANN-40), and T_p_ and T_c_ also increased to different degrees under treatment conditions, indicating that quinoa starch after treatment had a higher gelatinization temperature. It should be noted that an increase in ANN temperatures reflected a shift in the gelatinization transition but did not by itself prove improved thermal stability during processing. The changes in ΔH, which decreased for most HMT samples and for ANN-50 and ANN-60 but increased for ANN-30 and ANN-40, indicated that the energy required for gelatinization was modified, which may reflect partial disruption or reinforcement of ordered structures, respectively. The increase in T_o_, T_p_ and T_c_ values was also reported for annealed potato starch after annealing treatment at 55 °C for 8 cycles in a previous study [32].

### 3.4. Analysis of Pasting Characteristics

As shown in Table 3, HMT significantly decreased the PV, TV, BD, FV and SB values of quinoa starch as moisture content increased. The peak viscosity of QS was 1349.5 ± 4.95 cP, and when the HMT water content reached 30% (HMT-30), the viscosity decreased to 884 ± 33.94 cP. In contrast, ANN showed a temperature-dependent response: ANN-30 and ANN-40 increased several pasting parameters relative to native starch (PV: 1767.0 ± 118.8 cP for ANN-30, 1827.5 ± 30.4 cP for ANN-40 and 1349.5 ± 4.95 cP for native starch), while ANN-50 and ANN-60 decreased them (PV: 1022.0 ± 96.2 cP for ANN-50 and 1024.0 ± 9.9 cP for ANN-60). This phenomenon was also reported in yam starch, where the peak viscosity increased slightly after annealing at 30 °C but decreased after annealing at 40 and 50 °C [33]. Consistent with this, quinoa starch annealed at 50 °C exhibited lower peak viscosity than its native counterpart [33]. When the ANN temperature reached 60 °C (ANN-60), the peak viscosity reached the lowest value of 1024 ± 9.90 cP; such a change might be due to chain rearrangements in the amorphous region between the particles and the change in crystallinity during the treatment process, and this behavior was strengthened with the increase in HMT water content and ANN temperature. The gelatinization temperature of quinoa starch increased after HMT and ANN, indicating a shift in the gelatinization transition to higher temperatures, which was consistent with the DSC results. A negative SB value indicated that the final viscosity was lower than the trough viscosity, meaning that the paste viscosity continued to decrease during the cooling phase rather than increasing. This may be related to the breakdown of retrograded structures or the disruption of starch chain reassociation during cooling. The BD of HMT-30 and ANN-60 decreased to 170 ± 26.87 cP and 105.5 ± 2.12 cP, respectively. The reduction in BD values suggested that the treated starches were less susceptible to breakdown during continuous heating and shearing. Our findings on the effect of HMT on quinoa starch pasting properties were consistent with recent studies. Liu et al. reported that HMT at 20% and 30% moisture significantly decreased the viscosity and swelling power of quinoa starch, and that these changes were dependent on treatment moisture level [34].

### 3.5. Long-Range Ordered Structure Analysis

**Figure 2:**
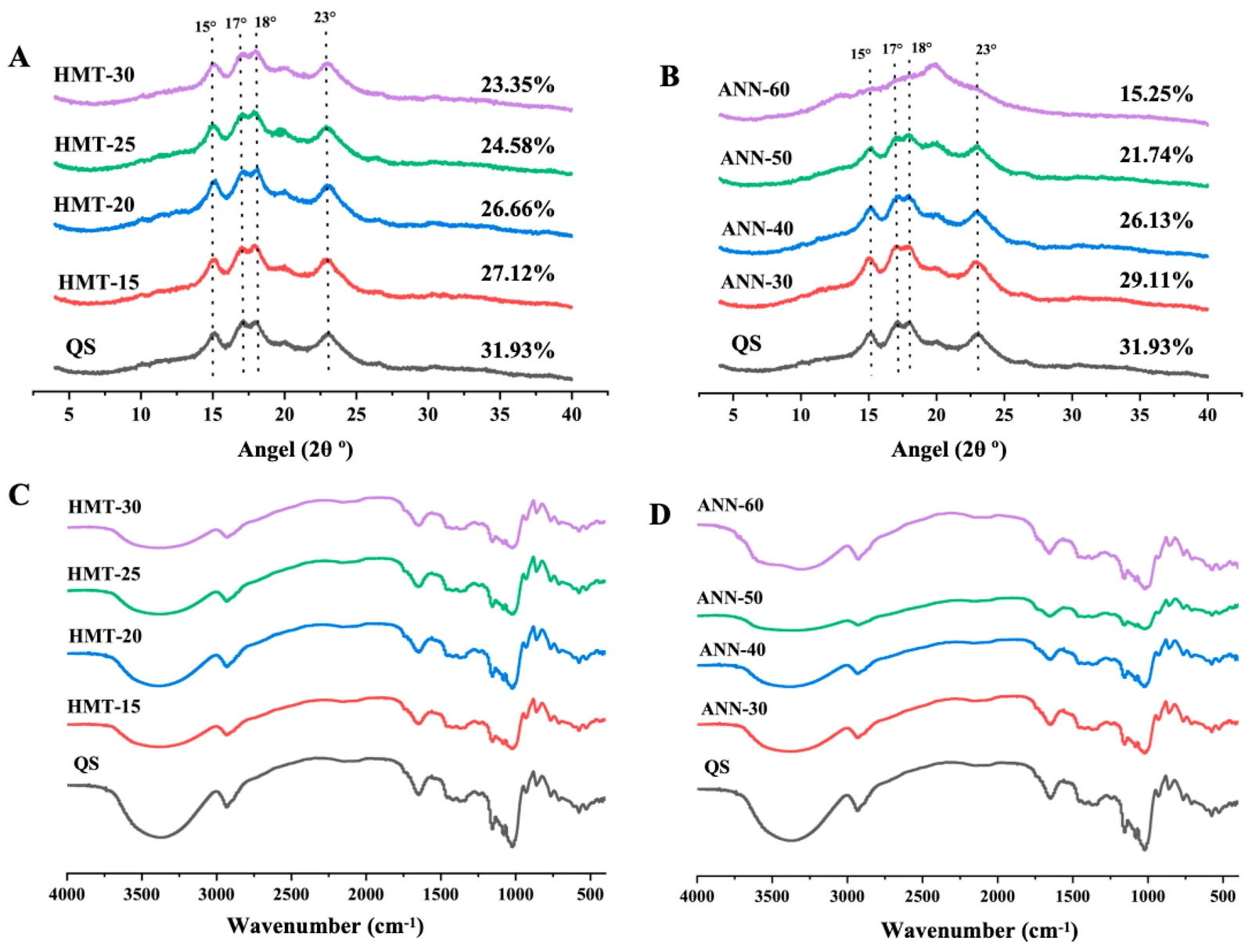
XRD patterns (**A**,**B**) and FT-IR spectra (**C**,**D**) of quinoa starch before and after HMT and ANN treatments.

The changes in starch crystal type and relative crystallinity (RC) of quinoa starch under HMT and ANN treatments were characterized by XRD. Figure 2A,B shows XRD patterns of quinoa starch before and after ANN and HMT treatments. The XRD pattern of the native quinoa starch showed a typical A-type crystal structure at 15°, 17°, 18° and 23°, which was consistent with previous findings for quinoa starch reported by Gu et al. [35]. The characteristic peaks at 17° and 18° decreased with the increase in water moisture and annealing temperature, which might be due to the destruction of the ordered structure of starch by moisture and heat during the treatment [6]. A similar phenomenon was also reported in proso millet starch with HMT and ANN treatments [25].

The RC of quinoa starch decreased to different degrees under different treatment conditions. For HMT, RC decreased from 31.93% (QS) to 23.35% (HMT-30) as moisture content increased. For ANN, RC decreased from 31.93% (QS) to 15.25% (ANN-60) as annealing temperature increased. However, the reported changes in crystallinity after ANN treatment of quinoa starch in previous studies were inconsistent. Chen et al. [5] conducted a 5-day annealing treatment of quinoa starch (20%, *w*/*v*) at a temperature 14 °C below the onset temperature of gelatinization (approximately 47 °C), and found no significant change in RC, attributing it to the higher proportion of short B chains in the branched starch of quinoa, which restricted the correct arrangement of the double helix in the crystalline region. We speculated that the difference in the results might be due to the small size (0.4–2.0 μm) of the quinoa starch granules [36], which have a large surface area and are more sensitive to thermal disturbances [37]. Additionally, the high proportion of short chains may result in thin and irregular crystalline layers. During annealing, the chain segments are prone to slip off and reorient rather than complete stacking, thus resulting in different outcomes under the same conditions [5].

### 3.6. Short-Range Ordered Structure Analysis

As shown in Figure 2C,D, no obvious new absorption bands were detected under the applied FTIR conditions, indicating that no new functional groups or covalent bonds were formed within the detection limit of conventional FTIR [38]. At the absorption wavelength of 3000 cm^−1^, the absorbance fluctuated greatly, which was related to the stretching vibration of O-H [39]. The intensity difference at 1047 cm^−1^ and 1022 cm^−1^ was related to the ordered crystallization and amorphous structure of starch, and the absorbance ratio R_1047/1022_ at 1047cm^−1^ and 1022cm^−1^ was used to characterize the short-range ordering degree of starch [40]. According to Table 4, the R_1047/1022_ values of quinoa starch treated with HMT and ANN were lower than that of the untreated quinoa starch, indicating that the short-range ordered structure of the starch was damaged to some extent during the treatment. The decrease in R_1047/1022_ after HMT and ANN treatment was consistent with the decreased crystallinity degree determined by XRD.

### 3.7. Starch Component Analysis

**Figure 3:**
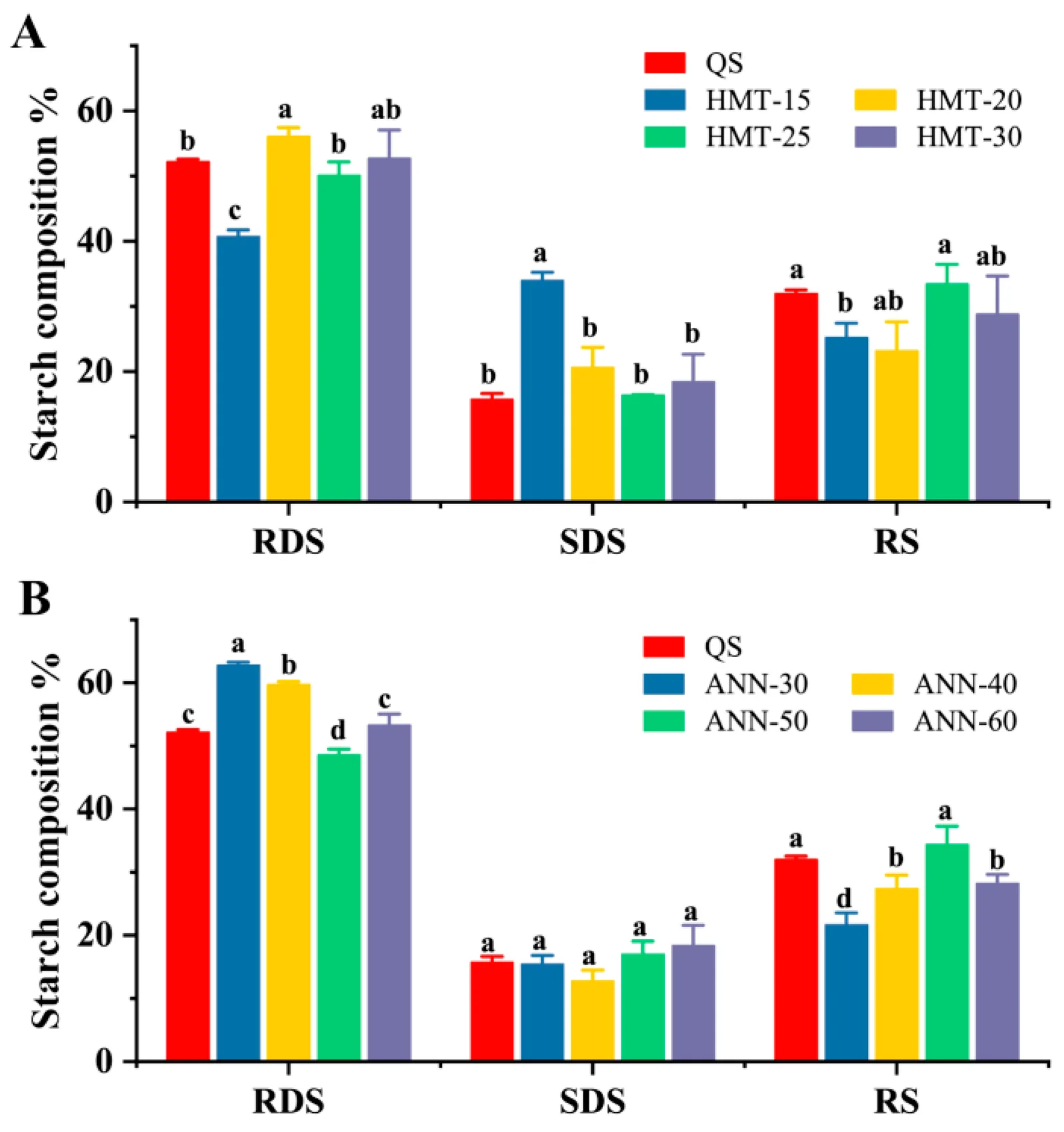
Starch composition (%) of quinoa starch samples with HMT (**A**) and ANN (**B**) treatments. Different lowercase letters within the same group indicate statistically significant differences (*p* < 0.05).

Figure 3 shows the RDS, SDS, and RS proportions of quinoa starch before and after HMT and ANN. The contents of RDS, SDS and RS in quinoa starch before treatment were 52.21 ± 0.36%, 15.78 ± 0.86% and 32.01 ± 0.50%, respectively. In the HMT group, RDS content first decreased and then increased as moisture content increased. At 15% moisture, RDS was significantly reduced to 40.77 ± 0.96% (*p* < 0.05), and SDS was significantly increased to 34.05 ± 1.23% (*p* < 0.05). This might be due to the increase in water in the system, which resulted in the gelatinization of hydrated starch, the overflow of amylose from the particles, and the recovery of starch during the cooling stage, thereby further decreasing the digestibility of starch [40]. At 20–30% moisture, RDS showed no significant change compared with native starch (*p* > 0.05). Compared with the previous XRD and FTIR data, it could be seen that the crystal structure and double helix structure of quinoa treated by HMT became worse, making it more accessible to enzymes. Liu et al. [1] and Dong et al. [41] reported that quinoa starch treated with HMT significantly decreased RDS while increasing SDS, with the highest SDS content. Our findings were consistent with these studies in demonstrating that HMT reduces RDS and increases SDS/RS. However, we additionally identified a moisture-dependent threshold effect—15% moisture optimally increased SDS to 34.05% and reduced RDS to 40.77%, whereas higher moisture levels lost this benefit. This threshold effect has not been previously reported for quinoa starch.

In the ANN group, ANN-30 exhibited the highest RDS content (60.79 ± 0.48%), indicating that low-temperature annealing promoted the formation of rapidly digestible starch. As the annealing temperature increased to 40 °C, the RDS content slightly decreased, but it was still significantly higher than that of native starch. In contrast, the RDS content of ANN-50 was significantly lower than that of native quinoa starch (*p* < 0.05), suggesting that a higher annealing temperature was conducive to the formation of slow digestion properties. This trend was consistent with the results of Liu et al.’s study [23], who reported that after 24 h annealing at 50 °C for quinoa starch, the total hydrolysis rate and RDS were significantly reduced. However, our results further revealed a temperature-dependent transition: ANN-30 unexpectedly increased the RDS content, while ANN-50 reduced it. This contrast indicated that ANN’s effect on the digestibility of quinoa starch is not monotonous, but depends on whether the treatment temperature is high enough to induce molecular rearrangement.

Based on the results of the digestion and changes in starch structure, we hypothesized that ΔH of HMT-15 decreases while To, Tp, and Tc increase. This indicated that under limited moisture (15%) and high temperature (100 °C), some unstable crystalline regions are melted and destroyed. During the cooling process, the molecular chains rearrange, and the remaining crystalline regions become more complete and stable, making it difficult for the enzyme to access. Therefore, the RDS is the lowest (40.77%). For ANN-50, ΔH only slightly decreases while To, Tp, and Tc increased significantly. This indicated that 50 °C was below the gelatinization onset temperature (61.4 °C), so no gelatinization occurs. However, the excessive moisture enabled the molecular chains to have sufficient mobility, and prolonged incubation causes some of the unstable crystalline regions to be partially destroyed and the remaining crystalline regions to rearrange more stably. This also reduced the accessibility of the enzyme, resulting in a RDS of 48.57%. In contrast, for ANN-30, ΔH increased while To, Tp, and Tc decreased. This suggested that at 30 °C, the molecular mobility was insufficient, and no effective rearrangement occurred. Instead, new, unstable local ordered structures were formed, which may be located on the particle surface or in areas more accessible to the enzyme. This increased the accessibility of the enzyme, leading to an increase in RDS.

**Figure 4:**
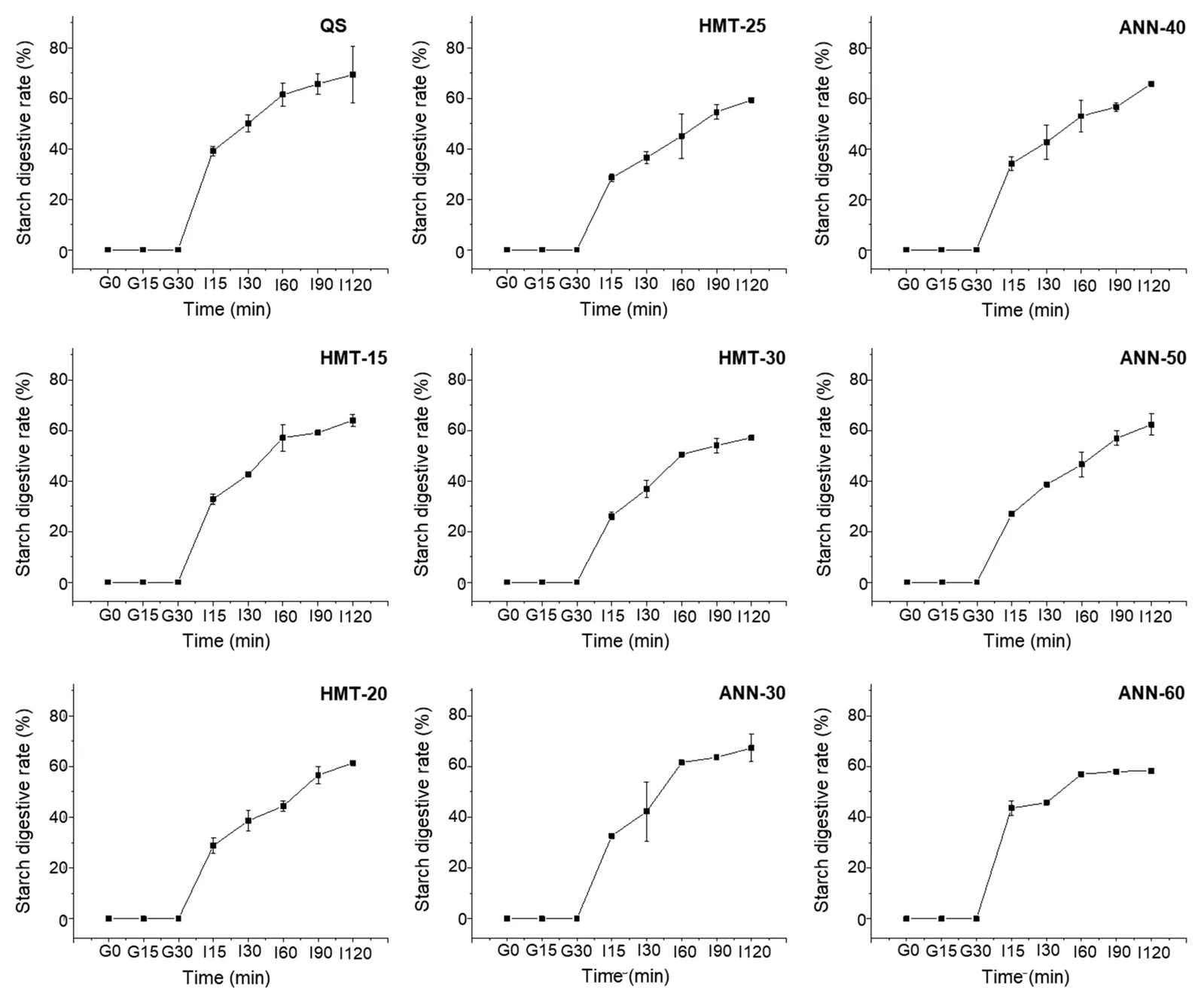
In vitro digestive rate of quinoa starch treated with HMT and ANN.

As depicted in Figure 4, during the simulation of digestion in the stomach, almost no quinoa starch was digested before and after the treatment, which might be related to the lack of digestive enzymes capable of breaking down the starch in the strong acidic environment of the simulated gastric fluid. During the simulation of digestion in the small intestine, due to the presence of a large amount of pancreatic amylase, invertase, and saccharifying enzymes, the quinoa starch was rapidly digested. After 120 min of digestion in the small intestine, the native quinoa starch was digested by 69.33%, while the quinoa starch treated with HMT was digested by 63.97% (HMT-15), 61.42% (HMT-20), 59.24% (HMT-25), and 57.06% (HMT-30), and the quinoa starch treated with ANN was digested by 67.29% (ANN-30), 65.73% (ANN-40), 62.27% (ANN-50), and 58.20% (ANN-60). After treatment under different conditions, the digestibility of quinoa starch showed a gradually decreasing trend as the moisture content of the HMT system increased and the ANN temperature rose.

A decrease in cumulative digestion at 120 min indicated that a larger proportion of starch remained undigested, which was consistent with an increase in RS content or a shift from RDS to SDS. In the present study, HMT-15 showed the highest SDS content (34.05%) and a relatively low cumulative digestion (63.97%), while HMT-30 showed the lowest cumulative digestion (57.06%) despite no significant change in RDS, SDS, or RS, suggesting that the undigested fraction at 120 min may include both RS and SDS that continue to hydrolyze beyond 120 min.

## 4. Conclusions

This study systematically compared the effects of HMT and ANN treatment on the physicochemical properties and digestibility characteristics of quinoa starch, revealing the different structural reorganization methods and functional regulation caused by the differences in treatment environments. For quinoa starch, this study conducted a systematic comparison across moisture contents (15–30%) and temperatures (30–60 °C), clarifying the quantitative relationship between treatment conditions and digestibility. This study found that the regulation of digestibility by HMT exhibits a water-dependent threshold effect—only 15% water HMT can effectively reduce RDS and increase SDS content, while higher moisture levels lose this effect; the effect of ANN was limited and temperature-dependent, low-temperature ANN (30 °C) instead increased RDS content, and high-temperature ANN (50 °C) decreased RDS content, while ANN-60 showed no significant effect on RDS content. This threshold effect and temperature transition had not been reported before in quinoa starch, providing clear evidence for the targeted selection of HMT moisture content and ANN temperatures to customize the digestibility of quinoa starch and develop low-glycemic index foods.

## Figures and Tables

**Table 1:** Water evolution rate (%) of quinoa starch with HMT and ANN treatments.

Sample	Water Evolution Rate
QS	30.19 ± 0.22 ^d^
HMT-15	36.40 ± 1.89 ^c^
HMT-20	35.22 ± 0.67 ^c^
HMT-25	40.64 ± 0.56 ^b^
HMT-30	42.22 ± 0.78 ^a^
ANN-30	31.60 ± 1.11 ^d^
ANN-40	34.83 ± 1.67 ^c^
ANN-50	42.30 ± 0.12 ^a^
ANN-60	42.92 ± 0.22 ^a^

Note: Different letters in the columns represented significant difference (*p* < 0.05).

**Table 2:** Thermal parameters of quinoa starch with HMT and ANN treatments.

Sample	ΔH (J/g)	T_o_ (°C)	T_p_ (°C)	T_c_ (°C)
QS	3.73 ± 0.46 ^bc^	61.36 ± 0.21 ^ef^	69.06 ± 0.41 ^cd^	76.76 ± 0.81 ^de^
HMT-15	3.23 ± 0.27 ^cd^	62.44 ± 0.52 ^de^	69.77 ± 0.19 ^c^	77.77 ± 2.65 ^cde^
HMT-20	2.54 ± 0.33 ^e^	62.91 ± 0.11 ^cd^	69.70 ± 0.07 ^c^	75.57 ± 0.18 ^e^
HMT-25	2.42 ± 0.17 ^e^	64.34 ± 1.61 ^c^	69.41 ± 1.00 ^c^	79.96 ± 1.80 ^bc^
HMT-30	2.87 ± 0.04 ^de^	63.30 ± 0.30 ^cd^	70.08 ± 0.49 ^c^	79.86 ± 0.51 ^bc^
ANN-30	4.72 ± 0.06 ^a^	58.40 ± 0.16 ^g^	67.17 ± 0.08 ^e^	76.57 ± 0.01 ^de^
ANN-40	4.15 ± 0.04 ^b^	60.54 ± 0.08 ^f^	68.05 ± 0.06 ^de^	79.47 ± 0.16 ^bcd^
ANN-50	3.60 ± 0.05 ^c^	68.23 ± 0.29 ^b^	71.90 ± 0.25 ^b^	81.05 ± 0.47 ^b^
ANN-60	2.86 ± 0.02 ^de^	76.07 ± 0.17 ^a^	79.04 ± 0.29 ^a^	86.60 ± 1.32 ^a^

Note: Different letters in the columns represented significant difference (*p* < 0.05).

**Table 3:** Pasting characteristics of quinoa starch with HMT and ANN treatments.

Sample	PV (cP)	TV (cP)	BD (cP)	FV (cP)	SB (cP)	PT (°C)
QS	1349.5 ± 5.0 ^b^	991.5 ± 13.4 ^c^	358.0 ± 18.4 ^a^	982.0 ± 9.9 ^c^	−9.5 ± 3.5 ^a^	94.7 ± 0.0 ^a^
HMT-15	1123.5 ± 31.8 ^c^	926.5 ± 20.5 ^cd^	197.0 ± 11.3 ^b^	899.0 ± 21.2 ^cde^	−27.5 ± 0.7 ^b^	92.6 ± 0.3 ^e^
HMT-20	1019.5 ± 26.12 ^c^	829.5 ± 20.5 ^e^	190.0 ± 5.7 ^b^	801.5 ± 21.9 ^f^	−28.0 ± 1.4 ^b^	93.3 ± 0.2 ^cd^
HMT-25	1029.0 ± 2.8 ^c^	841.5 ± 6.4 ^de^	187.5 ± 3.5 ^b^	813.0 ± 5.7 ^f^	−28.5 ± 0.7 ^b^	93.1 ± 0.0 ^de^
HMT-30	884.0 ± 33.9 ^d^	714.0 ± 7.1 ^f^	170.0 ± 26.9 ^b^	707.0 ± 7.1 ^g^	−7.0 ± 0.0 ^a^	93.9 ± 0.0 ^bc^
ANN-30	1767.0 ± 118.8 ^a^	1368.0 ± 55.2 ^b^	399.0 ± 63.6 ^a^	1249.5 ± 51.6 ^b^	−118.5 ± 3.5 ^e^	93.9 ± 0.6 ^bc^
ANN-40	1827.5 ± 30.4 ^a^	1466.0 ± 28.3 ^a^	361.5 ± 2.2 ^a^	1373.0 ± 28.3 ^a^	−93.0 ± 0.0 ^d^	92.9 ± 0.3 ^de^
ANN-50	1022.0 ± 96.2 ^c^	995.5 ± 94.2 ^c^	26.5 ± 2.1 ^d^	952.5 ± 95.5 ^cd^	−43.0 ± 1.4 ^c^	94.1 ± 0.3 ^ab^
ANN-60	1024.0 ± 9.9 ^c^	918.5 ± 12.0 ^cde^	105.5 ± 2.1 ^c^	876.0 ± 12.7 ^def^	−42.5 ± 0.7 ^c^	94.1 ± 0.3 ^ab^

Note: Different letters in the columns represented significant difference (*p* < 0.05). Peak viscosity (PV), trough viscosity (TV), breakdown (BD), final viscosity (FV), setback (SB), and pasting temperature (PT).

**Table 4:** The RC (%) and R_1047/1022_ of quinoa starch with HMT and ANN treatments.

Sample	RC	R_1047/1022_
QS	31.93 ± 0.32 ^a^	1.22 ± 0.06 ^a^
HMT-15	27.12 ± 0.68 ^c^	1.14 ± 0.08 ^ab^
HMT-20	26.66 ± 0.51 ^c^	1.10 ± 0.04 ^b^
HMT-25	24.58 ± 0.37 ^d^	1.08 ± 0.01 ^b^
HMT-30	23.35 ± 0.42 ^d^	1.07 ± 0.02 ^b^
ANN-30	29.11 ± 0.84 ^b^	1.22 ± 0.05 ^a^
ANN-40	26.13 ± 0.65 ^c^	1.17 ± 0.07 ^ab^
ANN-50	21.74 ± 0.87 ^e^	1.10 ± 0.02 ^b^
ANN-60	15.25 ± 0.53 ^f^	1.06 ± 0. 04 ^b^

Note: Different letters in the columns represented significant difference (*p* < 0.05).

## Data Availability

The data presented in this study are available on request from the corresponding author.
